# Dilution Is Not Always the Solution: A Retrospective Study of Pulmonary Lavage in Inhalation Injury

**DOI:** 10.1093/jbcr/iraf078

**Published:** 2025-05-08

**Authors:** Ashleigh Bull, Colette Galet, Samuel W Jones, Alexander Kurjatko

**Affiliations:** Department of Surgery, Division of Acute Care Surgery, University of Iowa, Iowa City, IA, United States; Department of Surgery, Division of Acute Care Surgery, University of Iowa, Iowa City, IA, United States; Department of Surgery, Division of Acute Care Surgery, University of Iowa, Iowa City, IA, United States; Department of Surgery, Division of Acute Care Surgery, University of Iowa, Iowa City, IA, United States

**Keywords:** bronchoscopy, inhalation injury, lavage, pulmonary lavage, complications

## Abstract

Burned patients with inhalation injury commonly undergo bronchoscopy, at times with pulmonary lavage (PL). We characterized the outcomes of burned patients with inhalation injury who underwent PL at a single burn center in a retrospective cohort study. We included all adult patients admitted between July 1, 2015 to June 30, 2023 who were on the ventilator and diagnosed with inhalation injury. Chemical inhalation, grade 0 inhalation injury, and diagnosis of inhalation injury without bronchoscopy were excluded. Demographics, burn size and anatomic location, and hospital course information were collected. Chi-square and Fisher’s exact tests were used to compare categorical variables, and continuous variables were compared using the Mann-Whitney *U* test. Multivariate analyses were performed to identify variables associated with outcomes. *P* < .05 was considered significant. One hundred sixteen patients were included; 37 (31.9%) underwent PL. Univariate analysis showed no significant differences in age, total body surface area burned (TBSA) second or third degree TBSA, complication rates, or in-hospital mortality between the no-PL and PL groups. Patients in the PL group had increased ventilator days (6 [2.5-15.5] vs. 2 [1-6], *P* < .001) and hospital length of stay (LOS) (12 [4-37.5] vs. 5 [2-18], *P* = .003). Multivariate analysis showed that PL was associated with an increase in ventilator days (OR = 1.84 [1.14-2.98], *P* = .013), hospital LOS (OR = 1.717 [1.080-2.730], *P* = .022), and sepsis (OR = 7.216 [1.106-47.080], *P* = .039). In conclusion, PL was associated with longer ventilator days, longer LOS, and increased risk of sepsis.

## INTRODUCTION

Bronchoscopy is a standard part of burn admission protocols for intubated patients. Lavage during bronchoscopy may be performed for a variety of reasons, such as clearing mucous plugs and gathering admission cultures. Experts recommend bronchoscopy and serial bronchoscopy in the evaluation and management of patients with inhalation injury.^1^ However, the therapeutic value of pulmonary lavage (PL) is unclear, and the techniques are not standardized.

Huber and colleagues used saline lavage with large volumes to induce acute respiratory distress syndrome (ARDS) in mongrel dogs, with negative changes observed in the alveolar architecture and loss of pulmonary surface activity.^2^ Saline lavage is used in many animal models, including guinea pigs and mice, to mimic ARDS as repeated lavage reduces surfactant lipid concentration and changes the alveolar surface tension.^3,4^ However, an animal study on inhalation injury revealed a reduction in free radical metabolites and inflammatory mediators in dogs who underwent high volume PL.^5^

In humans, serial bronchoscopy was found to improve oxygenation and time on mechanical ventilation as well as intensive care unit (ICU) stays among patients with severe inhalation injury.^6^ A prospective study in human subjects demonstrated no difference in pneumonia rates, but lower overall morbidity among patients with inhalation injury undergoing serial bronchoscopy with lavage for culture collection and airway clearance. The same study demonstrated a trend toward decreased ventilation and ICU days, and hospital length of stay (LOS) for patients receiving serial bronchoscopy, though this was not statistically significant.^7^

As most patients with a suspected inhalation injury undergo bronchoscopy on admission, it is important to understand how irrigation of the lung fields may clinically impact patients. This retrospective study aimed to review patients who had received PL during their admission and characterize their outcomes, with the hypothesis that there would be an association of clinical benefit with undergoing PL.

## METHODS

### Ethical statement

Our Institutional Review Board approved this retrospective study (IRB No. 201712728). A waiver of consent was approved for all subjects.

### Study design

Our institution’s burn registry was queried retrospectively to identify all burn patients aged 18 and older admitted from July 1, 2015 to June 30, 2023 who were on the ventilator and diagnosed with inhalation injury. Chemical inhalation, grade 0 inhalation injury, and diagnosis of inhalation injury without bronchoscopy were excluded. This study follows the Strengthening the Reporting of Observational Studies in Epidemiology reporting guidelines for cohort studies.^8,9^

### Institutional practice

Adult patients admitted to the burn unit on the ventilator with inhalation injury underwent diagnostic fiberoptic flexible bronchoscopy upon admission. At the attending physician’s discretion, PL was performed with isotonic normal saline to remove soot and debris from the inner lining of the lungs. The bronchoscopy was performed by a physician; bronchoscopy was supervised by an attending physician when performed by a resident or fellow physician. There was no standardized protocol for the performance of PL based on grade, for the performance of serial bronchoscopies, nor for the volume of normal saline to accomplish PL. The decision to obtain a culture sample from the PL was at the discretion of the attending physician. When patients on the ventilator were suspected to have inhalation injury, they were treated with 100% FiO_2_ and given Cyanokit. They were treated with nebulized heparin, acetylcysteine inhalation, and albuterol while on the ventilator.

### Data collection

Demographics (age, sex, and race), comorbidities (smoker status, chronic obstructive pulmonary disease [COPD], hypertension [HTN], diabetes mellitus [DM], obesity, and steroid use), hospital course (hospital LOS, ventilator days), injury information (mechanism of injury, presence of inhalation injury, total burn surface area [TBSA], second degree burn surface area, third degree burn surface area, burn injury body location), complications (ventilator-associated pneumonia [VAP], ARDS, sepsis, acute kidney injury [AKI], urinary tract infection [UTI], catheter-associated UTI [CAUTI], and central Line-associated bloodstream infection [CLABSI]), and mortality were obtained from our institution’s Burn Registry. All complications’ definitions follow the Burn Care Quality Platform data dictionary. Medical records were reviewed to identify patients who met our inclusion criteria. The following variables were also collected from medical records: use of PL, blood urea nitrogen >5, partial pressure of carbon dioxide (paCO_2_) >50, Richmond Agitation-Sedation Scale ≥5, initial carbon monoxide levels, use of hydroxy-cobalamin, and time from admission to the first bronchoscopy.

### Statistical analysis

Normality was assessed using the Kolmogorov-Smirnov test for all continuous variables. All non-normally distributed continuous variables are presented as median and interquartile range. Chi-square and Fisher’s exact test were used to compare categorical variables, while Mann-Whitney *U* test was used for continuous variables. Binary and negative binomial logistic regression analyses were performed to identify variables, including PL, associated with outcomes. Variables included in the initial models were age, sex, neck burn, inhalation injury grade, TBSA, and second-degree TBSA. All analyses were performed using SPSS 28.0 (IBM, Chicago, IL), and *P* < .05 was considered significant.

## RESULTS

### Patient selection

**Figure 1.**
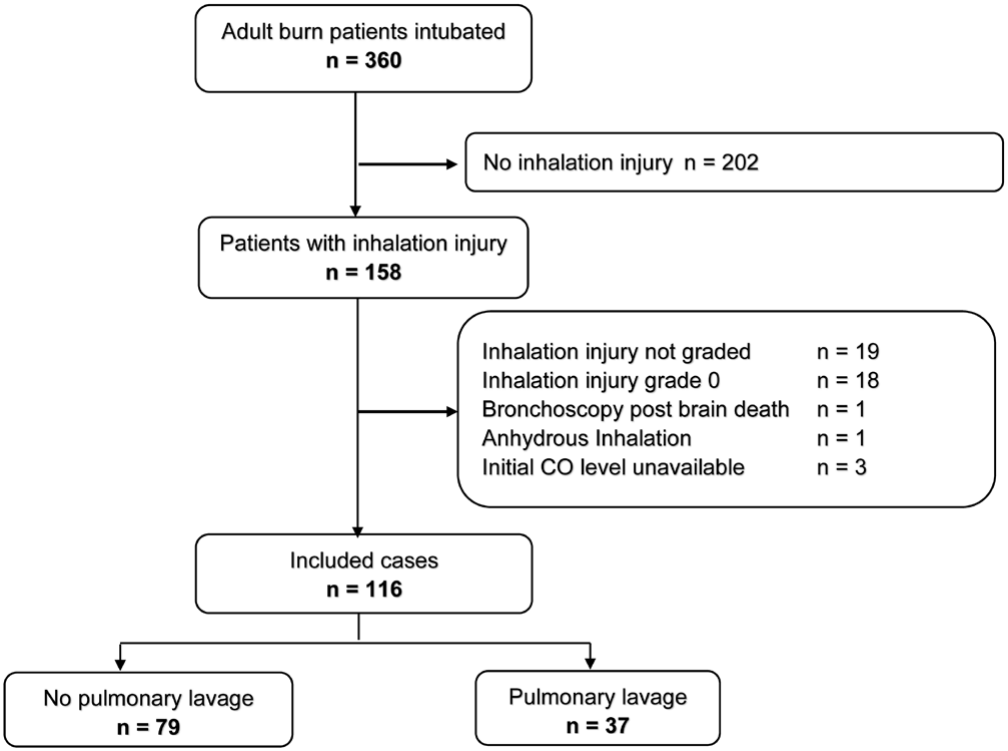
Patient Selection Flowchart.

As shown in **Figure 1**, 360 patients were identified in our institution’s burn registry as being mechanically ventilated on the burn unit for any length of time during the study period. A total of 158 patients had inhalation injury. After applying our inclusion and exclusion criteria, 116 were included in the study. Seventy-nine patients did not receive PL, and 37 received PL.

### Patient characteristics

As shown in **Table 1**, there was no significant difference between the groups in terms of demographics, comorbidities, and burn injury. Patients who received PL were less likely to present with COPD and/or a neck burn.

**Table 1.** Patient Characteristics

Variables	No pulmonary lavage	Pulmonary lavage	
	*n* = 79	*n* = 37	*P*-value
Male, *n* (%)	52 (65.8)	29 (78.4)	.198
Age, median [IQR]	57 [39-68]	55 [41-61.5]	.496
BMI, median [IQR]	27.1 [23.5-33.8]	27.4 [25.7-32.0]	.888
Comorbidities, *n* (%)	69 (87.3)	30 (81.1)	.406
Smoker, *n* (%)	36 (45.6)	16 (43.2)	.844
COPD, *n* (%)	28 (35.4)	6 (16.2)	**.048**
HTN, *n* (%)	38 (48.1)	14 (37.8)	.324
DM, *n* (%)	17 (21.5)	6 (16.2)	.621
Obesity, *n* (%)	33 (44)	11 (33.3)	.396
Steroid use, *n* (%)	3 (3.8)	0	.550
% TBSA, median [IQR]	11 [1-43]	11.5 [1.4-25.8]	.902
% second degree burn, median [IQR]	2.5 [0-12.6]	7 [1-15.9]	.061
% third degree burn, median [IQR]	0 [0-32]	0.2 [0-4.5]	.552
Burn location, *n* (%)			
Head	43 (54.4)	21 (56.8)	.844
Neck	29 (36.7)	6 (16.2)	**.030**
Anterior trunk	31 (39.2)	11 (29.7)	.408
Posterior trunk	32 (40.5)	20 (54.1)	.229
Facial burn below the nose, *n* (%)	37 (47.4)	13 (35.1)	.234
Inhalation injury with burn injury, *n* (%)	58 (73.4)	31 (83.8)	.248
Inhalation injury grade, *n* (%)			.056
1	55 (69.6)	19 (51.4)	
2	17 (21.5)	9 (24.3)	
3	7 (8.9)	9 (24.3)	
Culture, *n* (%)	21 (26.6)	26 (70.3)	<.001
Tracheostomy, *n* (%)	12 (15.2)	6 (16.2)	>.999
Need for skin grafting, *n* (%)	21 (26.6)	18 (48.6)	.022
Time from admission to wound closure (days), median [IQR]	17 [5.5-31.5]	23 [10.8-87]	.148

Bold indicates significant *P* values. Abbreviations: BMI, body mass index; COPD, chronic obstructive pulmonary disease; DM, diabetes mellitus; HTN, hypertension; IQR, interquartile range; TBSA, total burn surface area.

**Figure 2.**
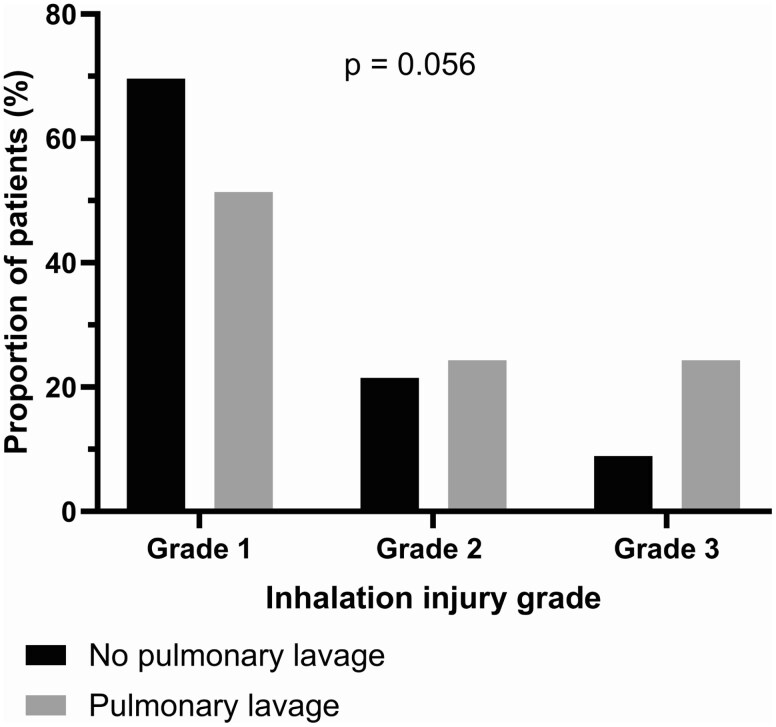
Proportion of Patients in the No Pulmonary Lavage and Pulmonary Lavage Groups Based on Inhalation Injury Grades.

There was no significant difference in the time from admission to bronchoscopy between the no PL and the PL groups (median = 157 [IQR = 81-303] min vs. median =221 [IQR = 79-599] min, *P* = 0.452). As shown in **Figure 2**, the proportion of patients with inhalation injury grade 2 and 3 tended to be higher in the PL group. Respiratory cultures were more often obtained in the PL group than in the non-PL group (70.6% vs. 26.6%, *P* < .001). There was no significant difference in the numbers of tracheostomy between the groups. In the PL group, 5 patients (13.5%) who required a tracheostomy had more than 1 PL; 3 had 2, 1 had 3, and 1 had 8 PLs. On average, tracheostomy was performed 16 days post-admission, ranging from 10 to 28 days, each indicated by the need for prolonged ventilation. Patients in the PL group were more likely to require skin grafting (48.6% vs. 26.6%, *P* = 0.022). For those who required skin grafting, the time from admission to wound closure was not significantly different between the groups (**Table 1**).

### Outcomes

Complication and mortality rates were similar between the groups, though the rate of sepsis complications in the PL group was twice that of the no PL group. Patients in the PL group stayed statistically longer on a ventilator and in the hospital (**Table 2**).

**Table 2.** Outcomes

Variables	No pulmonary lavage	Pulmonary lavage	
	*n* = 79	*n* = 37	*P*-value
Ventilator-associated pneumonia, *n* (%)	8 (10.1)	6 (16.2)	.370
Sepsis, *n* (%)	4 (5.1)	5 (13.5)	.142
Acute kidney injury, *n* (%)	7 (8.9)	4 (10.8)	.742
CAUTI, *n* (%)	4 (5.1)	1 (2.7)	>.999
CLABSI, *n* (%)	1 (1.3)	0	>.999
UTI, *n* (%)	1 (1.3)	1 (2.7)	.538
ARDS, *n* (%)	5 (6.3)	3 (8.1)	.709
Ventilator days, median [IQR]	2 [1-6]	6 [2.5-15.5]	**<.001**
Hospital length of stay, days; median [IQR]	5 [2-18]	12 [4-37.5]	**.003**
Mortality, *n* (%)	27 (34.2)	10 (27)	.524

Bold indicates significant *P* values. Abbreviations: ARDS, acute respiratory distress syndrome; CAUTI, catheter-associated urinary tract infection; CLABSI, central line-associated bloodstream infection; IQR, interquartile range; UTI, urinary tract infection.

### Multivariate analyses

As shown in **Table 3**, in binary logistic regression analyses, when adjusting for age, gender, inhalation injury grade, TBSA and second degree burn, PL was associated with an increased risk of sepsis (odd ratio [OR] = 7.216 [1.106-47.080], *P* = .039) but not with increased risk of VAP, ARDS, AKI, or mortality. Similarly, in negative binomial regression analysis, when adjusting for age, gender, inhalation injury grade, TBSA and second degree burn, PL was associated with an increased time on the ventilator (OR = 1.84 [1.14-2.98], *P* = 0.013) and increased LOS (OR = 1.717 [1.080-2.730], *P* = 0.022) (**Table 4**).

**Table 3.** Binary Logistic Regression Analyses

Variables	Sepsis			
	Odd ratio	95% C.I.		*P*-value
		Lower	Upper	
Age (y)	1.025	0.974	1.078	.341
Male	1.645	0.154	17.593	.681
Inhalation injury Grade (Ref = grade I)				.814
Grade 2	1.800	0.299	10.852	.521
Grade 3	0.000	0.000		.998
Second^-^degree burn	0.980	0.926	1.038	.494
TBSA	1.038	0.996	1.081	.076
Pulmonary lavage	7.216	1.106	47.080	**.039**

Bold indicates significant *P* values. Abbreviation: TBSA, total burn surface area.

**Table 4.** Negative Binomial Logistic Regression Analyses

Variables	Ventilator days				Hospital length of stay			
	Odd ratio	95% CI		*P*-value	Odd ratio	95% CI		*P*-value
		Lower	Upper			Lower	Upper	
Age (y)	1.007	0.992	1.022	.380	0.989	0.974	1.004	.137
Male	0.74	0.45	1.23	.246	0.528	0.328	0.851	**.009**
Neck burn	0.95	0.51	1.79	.879	1.16	0.64	2.13	.620
Inhalation injury Grade (Ref = grade 1)								
Grade 2	1.55	0.89	2.69	.120	1.03	0.61	1.73	.925
Grade 3	1.68	0.89	3.17	.111	1.59	0.86	2.94	.138
Second-degree burn	1.03	1.01	1.06	**.003**	1.039	1.016	1.062	**.001**
TBSA	1.01	1.00	1.02	.195	1.003	0.987	1.019	.730
Pulmonary lavage	1.84	1.14	2.98	**.013**	1.717	1.080	2.730	**.022**

Bold indicates significant *P* values. Abbreviation: TBSA, total burn surface area.

## DISCUSSION

Understanding how PL may clinically impact patients is important. Yet, the literature remains scarce. Currently, PL (including serial PL) is considered an appropriate treatment in the management of inhalation injury.^1^ Improved outcomes have been noted in some burned patients with pneumonia who underwent bronchoscopy compared with those who did not, with shorter duration of mechanical ventilation, shorter ICU and hospital stays, and most importantly, a reduced risk of death.^10^ Lavage does have observable benefits, such as the removal of casts or plugs that are obstructing the airway, causing immediate respiratory compromise.^11^ The true clinical effects of lavage as an adjunct to bronchoscopy remain unclear. Our study demonstrated poorer outcomes in patients who underwent PL in the setting of inhalation injury, even when adjusting for a grade of inhalation injury (Tables 3 and 4).

PL was associated with an increased risk of sepsis, though pneumonia rates were similar between the 2 groups. This may be related to cytokine expression in the pulmonary environment during inhalation injury. Interleukin-6 (IL-6) has been shown to be elevated in a large animal model with smoke inhalation.^12^ Cytokine expression patterns are an area of ongoing study in both sepsis and inhalation injury. In a prospective study performed on burn patients with inhalation injury aiming to quantify and characterize cytokines in bronchoalveolar lavage (BAL) specimens, IL-6 was easily measurable but not associated with any adverse outcomes in the final analysis. Other cytokines measured in the BAL samples were associated with adverse outcomes, suggesting the local environment at the alveolar level likely plays a role.^13^

The increased time on the ventilator observed in patients who underwent PL is concerning for a possible injurious effect on the pulmonary mechanics or pulmonary surface activity, leading to difficulty weaning from the ventilator. This is consistent with effects observed in large volume lavage that diluted and removed alveolar surfactant phospholipids to induce lung injury in animal models.^2,3^ This consideration has led to the evaluation of the use of pulmonary surfactant in the treatment of inhalation injury and its pulmonary sequelae.^14–17^ A Chinese study on rats exposed to inhalation injury demonstrated improved outcomes only in a group treated with the combination of saline lavage, pulmonary surfactant, and mechanical ventilation.^18^ Unfortunately, the utilization of surfactant in practice is inhibited by its high cost. On the other hand, in a retrospective review, Jiang et al. demonstrated both improved oxygenation and reduced duration of mechanical ventilation in patients undergoing bronchoscopy with lavage.^6^

Finally, we observed an increased LOS in patients undergoing PL; this is an expected result of increased time on the ventilator. Our findings are in contrast to a case series of 33 patients that demonstrated no difference in mechanical ventilation duration between patients who received serial bronchoscopies with lavage versus those who did not.^7^ While LOS in burned patients is certainly multifactorial, related to wound and surgical care, these patients were similar in burn size and severity. Despite the increased risk of sepsis, LOS, and duration of mechanical ventilation observed in our population, we did not see an increase in the presence of other important outcomes in inhalation injury, such as ARDS, pneumonia, and in-hospital mortality.

Our study presents several limitations. As a single center, retrospective study with a small sample size, we are limited to the data available in medical records. Only associations can be made from the data; no causality can be established. Only 3 patients had multiple bronchoscopies with lavage, which did not reveal any additional associations due to the small size. PL sample culture data were not available for these patients, limiting our ability to detect pulmonary infection that may have led to sepsis. At our center, we do not use high-frequency ventilation for inhalation, injury which is a suggested therapy. In our retrospective methodology, we do not have a reliable method for assessing the quality or frequency of hygiene measures, such as suctioning, that were performed. These may contribute to confounders that we cannot account for. Additionally, there is no standardized protocol for PL at our institution. Saline volumes administered between patients are variable and are not documented in the electronic medical record. The technique for lavage, such as the selection of bronchi and the decision to perform PL, is provider dependent at our institution, introducing additional variability into patient selection. Despite these limitations, our study provides insights that, despite the temptation to irrigate soot away during bronchoscopy, doing so may lead to worsened outcomes.

## CONCLUSION

Our study demonstrated increased ventilator days, hospital LOS, and risk of sepsis associated with undergoing PL. This was a limited study due to sample size, the constraints of utilizing a registry, and variability in practice surrounding PL at our burn center. A multicenter prospective study would be warranted to further evaluate the impact of a standardized PL protocol in inhalation injuries.
